# Type A and Type B personality among Undergraduate Medical Students: Need for psychosocial rehabilitation

**DOI:** 10.12669/pjms.306.5541

**Published:** 2014

**Authors:** Aliya Hisam, Mahmood Ur Rahman, Syed Fawad Mashhadi, Ghulam Raza

**Affiliations:** 1 Aliya Hisam, MBBS, MPH, Lecturer in Community Medicine Department, Army Medical College, Rawalpindi, Pakistan.; 2 Mahmood Ur Rahman, MBBS, DPH, MPH, MSc, FCPS, Professor and Head of Community Medicine Department, Army Medical College, Rawalpindi, Pakistan.; 3 Syed Fawad Mashhadi, MBBS, MPH, MPhil, Senior Lecturer in Community Medicine Department, Army Medical College, Rawalpindi, Pakistan.; 4 Ghulam Raza, MBBS, MSc (Medical Administration), Vice Principal, Army Medical College, Rawalpindi, Pakistan.

**Keywords:** Medical, Students, Personality disorder

## Abstract

***Objectives:*** To find out the frequency of Type A and Type B personality among the students of Undergraduate Medical College. To find association between student year and personality type.

***Methods: ***A descriptive cross sectional study was conducted at Undergraduate Medical College, Rawalpindi from Sept. 2012 till Feb. 2013. Among 500 sample size, 100 students from each MBBS year were inducted by probability systematic sampling technique. After taking consent from the institute and students, data was collected on BECK anxiety inventory (BAI) questionnaire. According to BAI scale, students were identified as Type A or B personality. Data was analyzed using SPSS version 20. To find association between student year and personality type, Chi-square test of significance with 95% confidence level was used.

***Results:*** First, second, third, fourth and final year students had 5 (1%), 6 (1.2%), 11 (2.2%), (13 (2.6%) and 19 (3.8%) type A personality respectively. Among all the study participants (n=500), total number of type A was 54 (10.8%) and type B personality students were 446 (89.2%). Type A personality was 29 (11.6%) in female students (n=250) and 25 (10%) in male students (n=250). Association between student year and personality type was significant (p=0.010) at 95% confidence level.

***Conclusion:*** Type A personality students existed in every class and there was a gradual increase in the number of type A personality students from 1st year to final year in an undergraduate medical college of Rawalpindi. Significant association was observed in student year and type A personality.

## INTRODUCTION

For the first time, type A behaviour was described in 1959 by two cardiologists as ‘an action-emotion complex that can be observed in any person who is aggressively involved in chronic, incessant struggle to achieve many goals at the same time’.^1^

Research shows that emotional resilience and behaviour traits have a significant impact on cardiovascular diseases. The presence of hostile behaviour in particular has effect on heart disease and is attributed as a factor for it as early as 1930s. Type A and coronary artery disease has positive link and positive association with stress also.^2^

Type A behaviour pattern (TABP), characterized by time urgency, impatience, and hostility, has been traditionally reported to be associated with coronary heart disease since the 1950s. In a study, 13% of the reports describe injuries due to aggressive behaviour.^3^

Ambitious, rigidly organized, highly status conscious, impatient are usually Type A personality. High-achieving ‘workaholic^4^^’ ^are usually type A personality who multi-task, push themselves with deadlines, and hate both delays and ambivalence.^5^ On the other hand Type B personality includes people who live at a lower stress level and typically work steadily, enjoying achievements but not becoming stressed when they are not achieved. Furthermore, Type B personalities may have a poor sense of time schedule and can be predominately right brained thinkers.^6^

To judge the presence of type A personality, several researchers have used impatience, anger, work involvement, time urgency, job dissatisfaction and competitiveness grades.^7^ According to a research conducted in USA, the prevalence of personality disorders (PD’s) was 13.4% (SE, 0.7).^8^

Although personality disorder is quite a prevalent condition and it also has association with hypertension, heart diseases and depression^9^, limited research is available regarding personality disorder association with comorbid conditions. If appropriate and timely intervention regarding anger and hostility are done regarding behaviour patterns, risk of coronary disease can be substantially reduced.^10^

We really need to know the numbers of type A personality in our set-up. It is of outmost importance that we collect information from medical institutes regarding behaviour pattern of the undergraduate medical students (UGMS). Medical professionals are considered healers of the community so it is important that they are mentally healthy. Because of the country present situation and medical education stress, future doctors are under double stress. There is a need to evaluate their psychological health and personalities. UGMS are going to be the future remedial personnel and if they are not mentally healthy it will be unethical to locate them in health care centers. While collecting the frequency of type A personalities in a undergraduate (UG) medical institute, awareness will also be created among UGMS regarding type A behaviour patterns and its negative health effects. Data regarding the type A personality frequency among UGMS, can later act as a data bank to find the relation of type A personality and coronary artery disease (CAD).

## METHODS

A descriptive cross sectional study was conducted at Undergraduate Medical College, Rawalpindi of 6 months duration from September 2012 till February 2013. Five hundred sample sizes was calculated by using WHO sample size calculator with confidence level of 95%, anticipated population proportion 0.134 and absolute precision of 0.03. Verbal informed consent from students and permission from the Ethical Committee of Undergraduate Medical College was taken. About 100 students from each MBBS year (50 males and 50 females) were inducted by probability systematic sampling technique. A sampling frame (separate list of male and female students from 1^st^ year MBBS to 5^th^ year MBBS) was obtained from the college administration. Those students under observation for some medical disease were excluded from the study list. Student strength of each MBBS year was 200. One hundred sample sizes was the requirement from each year having 200 student strength. The first sample was obtained from both lists (male and female sampling frame) by simple random sampling (lottery method). Then afterwards, every 2^nd^ individual (200/100=2) was selected from each list till the required sample size i.e. 100 was achieved per MBBS year (50 males and 50 females). Data was collected on BECK anxiety inventory (BAI) questionnaire.^11^ According to the criteria laid down by BECK interpretation scale^12^ students were scaled as follows:

**Table-I:** BECK Interpretation Scale^12^

**Range of Scores**	**Anxiety Level**
0-7	Minimal Level of anxiety
8-15	Mild Anxiety
16-25	Moderate Anxiety
26-63	Severe anxiety

**Table-II:** Frequency of type A and B personality among medical students (n=500)(p=0.010)

Student’s Year	Type A n (%)	Type B n (%)
1^st^ Year MBBS	5 (1)	95 (19)
2^nd^ Year MBBS	6 (1.2)	94 (18.8)
3^rd^ Year MBBS	11 (2.2)	89 (17.8)
4^th^ Year MBBS	13 (2.6)	87 (17.4)
5^th^ Year MBBS	19 (3.8)	81 (16.2)
Total	54 (10.8)	446 (89.2)

Then Type A and Type B was diagnosed according to the operational definitions. Score of 26 and above were defined as Type A personality while score of 25 and below were diagnosed below Type B personality. After filling the questionnaire from the student, they were diagnosed as having either type A or B personality.

Data was entered and analyzed using Statistical package for Social Sciences (SPSS) version 20. Qualitative data including variable such as MBBS year, gender and type of personality are presented in the form of frequencies and percentages. Chi-square test of significance with 95% confidence level is used to find association between MBBS student year and personality type.

## RESULTS

Among all the study participants (n=500), total number of type A was 54 (10.8%) and type B personality students were 446 (89.2%) as shown in Table-II. First year had 5 (1%) type A personality students and 95 (19%) type B personality students. Second year had 6 (1.2%) type A and 94 (18.8%) type B personality students. Third year had 11 (2.2%) type A and 89 (17.8%) type B personality students. Fourth year had 13 (2.6%) type A and 87 (17.4%) type B personality students. Fifth year had 19 (3.8%) type A and 81 (16.2%) type B personality students. Association between student year and personality type was significant (p=0.010) at 95% confidence level, as shown in Table-II. 

Out of the female students (n=250), 29 (11.6%)) were Type A and 221 (88.4%) were type B. Among male students (n=250), 25 (10%) were type A personality and 225 (90%) type B. Association between male and female type A personality was not significant (p=0.564) at 95% Confidence level.

## DISCUSSION

The number of type A personality is increasing in UGMS. Five hundred medical students were evaluated for behaviour pattern and increasing pattern of type A personality was seen year wise. Frequency of type A personality was observed more among female medical students as compared to male medical students. This is a concern and an important issue which must be thought into as many studies have concluded a positive relationship of type A personality and coronary artery disease. 

Undergraduate medical students in Pakistan use note-taking, reading textbooks or articles, organizing thoughts prior to writing, depending on the assortment of effective studying and learning methodologies. Medical teachers stretch their extent of information and knowledge in a logical, planned, integrated and sequential manner to the students through different approaches i.e didactic lectures, case base learning (CBL), clinical case scenarios, guided direction during tutorials, discussions and clarification of quiz answers. It in presumed that competency based education can be acquired by knowing prior knowledge of students, integrated teaching and use of multiple techniques and methods in teaching practice to promote confidence, understanding and test performance of students.^13^

Another study showed that the prevalence of Personality Disorders (PDs) is highest among subjects with only a high school education or less, and living without a partner in the center of the city.^8^ Eighty eight full-time doctoral students (males: 53, females: 35) in the age range of 26-55 (mean=35 years) were enrolled in a study for Type A behaviour Scale (TABS). They concluded that higher level of type A personality pattern is seen in female medical students as compared to males. Our study also concluded that female number of type A personality is more as compared to males i.e. 11.6% and 10% respectively. Second observation was that doctoral students that have spent 6 to 10 years in their studies presented higher levels of Type A behaviour pattern than those that spent 0-5 years; in this study, it was also observed year wise increase in type A personality. Third observation was that among older students (26-35 years) type A behaviour pattern was higher as compared to younger students (26-35 years). They also concluded that type A behaviour pattern positive individuals must be sensitized regarding its negative health consequences.^1^

A study on myocardial infarct group showed a significantly higher incidence of Type A personality in the myocardial infarction group.^2^ Our study was only focused on frequency of type A and B personality and year wise increase.

Another study concluded that sensitization of type A should be done as to the fact that the negative health effects associated with Type A behaviour**. **The Type A individuals should be taught to adopt healthier behaviour patterns with similarly successful outcomes. Type A behaviour causes high stress levels and can be moderated through exercise.^4^ After filling the questionnaire in our study, UGMS were given awareness regarding their personality type and association of type A personality with CAD, Injuries and other negative health effects.

Another study showed that a sub-group of Type A individuals are disliked by their co-workers.^3^ We did not include the element of relationship with other colleagues in our study objective as it is a very important aspect of medical profession. A study in China found that aggressiveness was a risk factor for unintentional injuries. Regarding proneness of accident, a case control study was conducted which showed association of TABP with accidents.^3^ Accidental history can be incorporated into this type of study which targets personality type evaluation. 

Another research showed in contrast to non-Type A patients, a significantly greater proportion of Type A patients had at least one artery with a clinically significant occlusion of 75% or greater.^10^ As our inclusion were UGMS, research studies regarding personality types at other end (CAD, Hypertensive, diabetics patient) can also be inducted to find the association of personality with other variables.

Important strength of our study was that we targeted every year of the medical students and large sample size 500. Students were selected by systematic randomized technique which increased the strength of the study as selection biased was reduced to a substantial level. This was a college based study and specifically targeting the UGMS with their recent state of mind so Berkosian bias, and/or recall bias has been reduced all together.

One of the most important limitations of our study was that we conducted it in only one undergraduate medical institute so results cannot be generalized. This study was a cross sectional study and we only had a snap shot of all years at one time. If possible, a cohort study shall be planned and conducted over at least a period of ten years as to find frequency with increasing years and type A personality. Another limitation was that we did not assess their socioeconomic status which would have highlighted the association between the socioeconomic status and type A personality.

This study is very beneficial for future research as to find a link between type A personality and coronary heart diseases, hypertension and depression, to ascertain the influence of personality factors on the course of coronary artery disease which is the major cause of deaths in our society.

## CONCLUSION

Type A personality students existed in every class of medical students and there was a gradual increase in the number of type A personality students from 1st year to final year. Female students were more type A personality as compared to males. There is a need for psychosocial rehabilitation in the medical colleges of Rawalpindi, Pakistan so as to encounter this minor mental health issue timely.

## Notes

***Source of Funding:*** Authors Personal Contribution.
